# What Have We Learnt about BCG Vaccination in the Last 20 Years?

**DOI:** 10.3389/fimmu.2017.01134

**Published:** 2017-09-13

**Authors:** Hazel M. Dockrell, Steven G. Smith

**Affiliations:** ^1^Faculty of Infectious and Tropical Diseases, Department of Immunology and Infection, London School of Hygiene and Tropical Medicine, London, United Kingdom

**Keywords:** BCG, vaccination, tuberculosis, efficacy, biomarkers, correlates of protection, immune responses, infants

## Abstract

A number of new tuberculosis (TB) vaccines have been or are entering clinical trials, which include genetically modified mycobacteria, mycobacterial antigens delivered by viral vectors, or mycobacterial antigens in adjuvant. Some of these vaccines aim to replace the existing BCG vaccine but others will be given as a boosting vaccine following BCG vaccination given soon after birth. It is clear that the existing BCG vaccines provide incomplete and variable protection against pulmonary TB. This review will discuss what we have learnt over the last 20 years about how the BCG vaccine induces specific and non-specific immunity, what factors influence the immune responses induced by BCG, and progress toward identifying correlates of immunity against TB from BCG vaccination studies. There is still a lot to learn about the BCG vaccine and the insights gained can help the development of more protective vaccines.

## Introduction

The BCG vaccine has been used since 1921 to prevent tuberculosis (TB) and is considered to be the world’s most widely used vaccine (1). Yet, it is well established that the protective efficacy of BCG varies depending in which geographical location it is administered and we understand very little about why it protects when it does, or why it fails to protect when it does not. This review will survey what we have learnt about BCG over the last 20 years. Although a lot of progress has been made, the past two decades have shown that there is no simple correlate of BCG-induced protection against TB even though T-cells and IFNγ are clearly required.

We still need to understand why BCG gives such variable protection against pulmonary TB in different settings. The BCG vaccine remains critical for the development of new TB vaccines, many of which will be given as a booster vaccine after BCG vaccination, and any factors that affect how BCG works may also impact on these as well as on new genetically modified BCG vaccines or other live mycobacterial vaccines (2, 3).

Neonatal BCG provides good protection against disseminated and pulmonary TB disease in young children (4) but variable efficacy against pulmonary TB in adults when given later in life. However, when it does protect, this protection can be long-lived, lasting for up to 15 years in the United Kingdom (5), 30–40 years in Norway (6), and even as long as 50–60 years in Alaska (7). There is also evidence from outbreak settings to support the hypothesis that BCG vaccination can protect against infection, as well as disease; for example, an association has been observed between the presence of a BCG scar and not only less disease but also lower rates of interferon-gamma release assay (IGRA) positivity with relative risks for vaccinated compared with unvaccinated children of 0.61 for infection and 0.51 for disease (8).

Over the last 20 years most of the world’s children have continued to receive BCG immediately after birth or when they are first in contact with health services. In 2005, the United Kingdom, which had previously vaccinated adolescents, switched to a targeted vaccination of infants at higher risk of infection (i.e., with parents or grandparents born in high incidence countries or who live in areas of the United Kingdom where the annual incidence of TB exceeds 40/100,000). Other countries have never given BCG, in order to retain the use of the tuberculin skin test (or the newer IGRA tests) as a means of assessing infection or because the risk of infection is low (9). BCG scars are often the best indication of prior vaccination that is available, but are an imperfect proxy for prior vaccination particularly if infants are vaccinated after birth (10).

Vaccination of young infants can, however, lead to disseminated BCGosis if the infant is HIV-infected (11). For this reason, the Global Committee on Vaccine Safety recommended that BCG vaccine should not be given to infants who were HIV infected. As HIV status is generally not known and as even in areas with higher HIV infection rates, most mothers will be on antiretroviral treatment, and their infants given post exposure prophylaxis, most infants are still being vaccinated without testing. As well as acquired immunodeficiencies, inherited genetic disorders of the immune system also confer susceptibility on infants to disseminated BCG infection following vaccination as well as to childhood TB [reviewed in detail here (12)]. A safer BCG for use in immunocompromised infants would therefore be beneficial.

## What have We Learnt about the Causes of Variable Protection Induced by BCG Over the Last 20 Years?

### Pre-Sensitization with Environmental Mycobacteria

BCG vaccination immediately or shortly after birth provides consistent protection against the disseminated forms of TB in young children (4, 13, 14), and is very cost effective (15). Neonatal BCG also protects against pulmonary TB in children (4) which contrasts with the variable protection seen against pulmonary TB in adolescents and young adults (16).

BCG vaccination is more protective against pulmonary disease in children and adults at higher latitudes and in those screened for prior sensitization by mycobacteria (4, 17). Environmental mycobacterial are often assumed to be the culprits, mycobacteria species that survive in different environmental niches, and colonize humans over time (18). The impact of prior sensitization, as detected by tuberculin skin testing, on the protective efficacy of BCG against pulmonary TB has been shown in a number of settings (4) but not whether this induces masking of the BCG vaccine effect (inducing protection that BCG cannot improve upon) or blocking (inducing pre-existing immune responses that prevent BCG vaccine-induced, protective responses) (19). One reason for limited progress has been the lack of antigens specific for environmental mycobacteria with which to dissect T-cell responses to the different mycobacteria other than tuberculosis or non-tuberculous mycobacteria (NTM; e.g., *Mycobacterium avium*) (20). Skin test positivity to *Mycobacterium tuberculosis* purified protein derivative (PPD or tuberculin) increases with age, although indurations >10 mm are less frequent in those who are BCG vaccinated (21). It is widely assumed that the effects of environmental mycobacteria or NTM would precede BCG vaccination, perhaps blocking BCG multiplication (22, 23); however, this is unlikely for infants vaccinated soon after birth, and too little work has focused on their effects post-BCG. In the mouse model, oral dosing with NTMs following BCG vaccination decreased protection (24).

### BCG Strain Variations

Genetic variability between the different BCG vaccine strains might explain variable protection. The original attenuated *Mycobacterium bovis* bacille Calmette Guerin was kept in culture for many years before it was properly conserved and seed lots were preserved by lyophilization. It was distributed to different locations worldwide where further culture resulted in what are now known as BCG Russia, Japan, Copenhagen, etc. Improved sequencing methods have shown that further genetic events have occurred in these strains, including additional genetic deletions (25, 26). Although some countries have only used one strain of BCG, in others, two or more strains have been used making analysis difficult (27). The different vaccine strains induce varying degrees of T-cell response in *in vitro* peripheral blood mononuclear cell (PBMC) cultures from those vaccinated with different BCG vaccine strains (19, 28). However, to date there has been no evidence that such genetic variation between strains accounts for variability in efficacy or that the protection given by a particular BCG vaccine strain is related to the year in which the study was performed (4).

### Route of Administration

BCG vaccine is delivered most often by the intradermal route. It can be given percutaneously by a multi-puncture device rather than intradermally but a large study in South Africa showed both delivery routes were equally protective (29, 30). Most data describing the impact of administration route on vaccine efficacy has come from animals. Originally BCG was given orally and studies in guinea pigs have shown that BCG can be as protective and with less pathology induced post-infection if given BCG is given orally (31). Giving both a BCG prime and an MVA85A boost to mice intranasally induced much more effective protection than seen using other routes of immunization (32), and more recently there is renewed interest in the mucosal route of vaccination (33). In rhesus macaques, improved BCG-induced protection was observed when the vaccine was administered endobronchially into the lungs when compared with parenteral vaccination (34).

There is also a body of data on the effect of administration route on the quantity and quality of the immune response induced. Percutaneous and intradermal routes induced similar immunogenicity in a study in South Korea (35). Giving BCG followed by the ID93 vaccine in GLA adjuvant intranasally induced tissue resident T-helper (Th)-17 T-cells rather than Th-1 T-cell immunity (36) and when BCG itself was given by the mucosal route, this resulted in effector memory and resident memory T-cells in the mouse lung (37).

### Vaccine Batch Variations

There have also been questions about viability or growth rate variations in different batches of vaccine, which are only required to have colony forming units between set limits (and thus may also contain varying numbers of dead bacilli). A study in West Africa found that batches of BCG showing slower growth were associated with larger BCG scars, and a higher prevalence of positive PPD skin test responses in the Mantoux skin test (38). Issues of batch or vaccine strain variability are very challenging to study at scale, as the EPI program will ship vaccine from one of several suppliers and thus, as mentioned above, many regions can have used three or more types of vaccine (9, 27), as well as different batches of the same vaccine strain.

### Global Differences in Non-Specific Protection?

There has been interest in the benefits of administering BCG for reasons other than as a vaccine against TB for many years. For example, BCG vaccine can be instilled into the bladder where it is an effective immunotherapy against non-muscle invasive bladder cancer (39, 40). BCG vaccination is also associated with reduced rates of asthma but despite a number of observational studies, analyzed in two meta-analyses (41, 42), there have only been two randomized clinical trials using neonatal BCG vaccination and only one trial showed some beneficial effect (43, 44).

Studies in West Africa have indicated that BCG vaccination may have beneficial effects on all-cause mortality in infants, although the effects were most marked in low birth weight infants (45). Until recently, these effects had not been examined in infants in higher income or latitude settings, so there was a lot of interest in a study in Copenhagen that hypothesized that giving BCG at birth to Danish infants would reduce the number of somatic infections. In 2,129 BCG vaccinated and 2,133 unvaccinated controls, BCG vaccination had no effect on all-cause hospitalizations [hazard ratio 1.05 (95% confidence intervals 0.93–1.18)] or on parent-reported infections up to the age of 15 months. There was no difference between outcomes in normal birth weight infants or premature infants in this European setting (46). Some of the previously described non-specific effects of vaccination show sex differences; however, in this Danish study, there was no difference in outcomes by sex (46). Thus, any non-specific effects may be more marked in certain low and middle-income country settings.

## Dissecting Immune Responses to BCG at the Cellular and Molecular Level

### Early Events following Vaccination

BCG is usually administered by intradermal injection. It has been possible, in animals, to investigate the vaccination site tissue for rapid innate immune responses induced by the array of pathogen-associated molecular patterns BCG is endowed with. These are largely components of the mycobacterial cell wall such as peptidoglycans, arabinogalactan, and mycolic acids that interact with Toll-like receptors (47) and other pattern recognition receptors such as complement receptor and mannose receptor as reviewed recently (48). The immediate response is characterized by inflammatory cytokines in BCG “lesions,” including IL-1β, TNFα, MCP-1, and IL-8, as demonstrated in rabbits. A biphasic pattern of expression indicated that the source of the cytokines is local resident innate cells (days 1–3) followed later by infiltrating mononuclear cells (day 9) (49). Although most studies of the immune response to BCG in humans focus on peripheral blood, punch biopsies at the vaccination site have revealed that live BCG persists until at least 4 weeks post-vaccination in previously unvaccinated adults. The cellular infiltrate at the vaccination site, as revealed by suction blister analysis included lymphocytes (CD3+) and monocytes (CD14+) but predominantly comprised CD15+ neutrophils (50). Interestingly, human blood neutrophils that have recently captured BCG mycobacteria *in vitro* have been shown to cooperate with dendritic cells to enhance antigen-specific T-cell responses (51). Despite the chronic nature of intracellular mycobacterial infections, neutrophils are also present in the immune response in human TB. Gene expression signatures associated with neutrophils were found in TB disease (52) and in the lungs of patients with TB, many neutrophils containing *M. tuberculosis* are present (53).

BCG lacks the *esx-1* locus which, although providing sufficient attenuation compared with *M. bovis* or *M. tuberculosis* to allow its use as a vaccine, prevents phagosomal rupture, cytosolic contact, and host cell death following uptake by phagocytic antigen presenting cells (54). As such, there are a host of cytosolic immune detection systems that are underused by the vaccine but which may represent pathways for the induction of more potently protective immune responses (54). Clearly, in the case of *M. tuberculosis*, some of these responses are responsible for enhanced virulence *via* increased inflammation, granuloma formation, and damage to lung structure and function however there appears to be an *esx*-related balance to be struck between pathogenicity and immunity. Recombinant BCG expressing ESX-1 from *M. marinum* had much reduced virulence in immunocompromised mice when compared with *M. tuberculosis* itself or BCG expressing ESX-1 from *M. tuberculosis* however immune responses and protection against *M. tuberculosis* challenge were improved when compared with BCG (55). Interestingly for the design of new vaccines for TB, the ESAT-6 related immune responses that are important for the control of *M. tuberculosis* rely, at least in part, on antigen-non-specific, IFNγ-producing CD8+ T-cells, and natural killer cells that are induced *via* the NLRP3-inflammasome-IL-18 pathway (56).

### Adaptive Immune Responses to BCG

The primary mechanism of action of BCG-induced protection against TB has long been thought to be Th-1 cell mediated and although the last 20 years have provided clues to the involvement of other effector mechanisms, the Th-1 paradigm remains in place. Resident dendritic cells travel to local lymph nodes bearing antigen/live BCG and activate antigen-specific CD4+ T-cells in the presence of type 1 polarizing cytokines such as IL-12 and IL-18. IFNγ-secreting T-cells therefore represent the canonical immune response to BCG vaccination (57). Despite robust Th-1 responses in adults following BCG, our understanding of the newborn immune system dictated that an equivalent response to BCG would be unexpected in infants who demonstrate a marked bias toward regulatory and Th-2 response in their natural reaction to microbial challenge. Surprisingly, BCG also proved to be a potent inducer of Th-1 responses in infants (58). The hypothesis that delaying BCG vaccination of infants might circumvent some of the more profound regulatory effects of the newborn immune response and lead to improved type 1 immunity has come in for some scrutiny, however study results have been mixed (see below).

The antigen specificity of BCG-induced T-cells is broad, reflecting the plethora of mycobacterial antigens possessed by BCG which are presented to the immune system (59). Dendritic cells that have collected BCG antigens have the ability to cross-present antigen and BCG-specific CD8+ T-cells are detectable following BCG stimulation of PBMC from BCG-vaccinated donors *in vitro* (60, 61). Improving this component of the BCG-induced vaccine response is one of several approaches being taken to enhance the immunogenicity of BCG and develop a better TB vaccine. One strategy has been the insertion of the Listeriolysin gene designed to facilitate access of BCG antigen to the endogenous antigen processing pathway (62); the BCG∆ureC:hly vaccine is more immunogenic in mice than parental BCG and induces Th-1 and IL-17-producing T-cells in man (63, 64). However, development of a recombinant BCG with a mutant Perfringolysin gene overexpressing Ag85A, Ag85B, and Rv3407 has been discontinued as varicella zoster virus was reactivated in 2/8 vaccinees, although the vaccine was immunogenic and induced anti-mycobacterial activity in man (65). Another approach is the use of adenovirus vectors rather than MVA to deliver *M. tuberculosis* antigens following BCG vaccination in a prime-boost regimen, as adenovirus vectors induce a more mixed CD4 and CD8 T-cell activation than MVA which is better at inducing CD4 T-cell responses (66).

One of the most prominent developments in the field of immunology in the last 20 years has been the rise of the Th-17 cell, initially identified in the context of autoimmune disease. Th-17 cell polarization is brought about by the activation of CD4+ T-cells in the presence of TGFβ and IL-6 and the Th-17 population is characterized by the expression of IL-17 and IL-22. Th-17 cells contribute to the granulomatous response to Mtb infection (67) and they are important for immune protection against more virulent, clinical isolates of *M. tuberculosis* (68). Th-17 cells were responsible for enhanced vaccine-induced protection against Mtb challenge in a mouse model (69, 70) and they are often taken as a sign of improved immunogenicity in pre-clinical models of novel TB vaccines. For example, the recombinant BCG vaccine BCG∆ureC:hly, which induces increased protection compared with parental BCG in mice, induces increased numbers of IL-17 producing helper T-cells (63), with some increase in CD8 T-cells producing IL-17 in human vaccinees (64). BCG is not considered a potent inducer of Th-17 cells. Our group detected IL-17+ CD4+ T-cells following infant BCG vaccination in the United Kingdom but at much lower frequencies than polyfunctional Th-1 cells (71). One explanation for poor Th-17 responses to BCG may be the strain’s lack of the RD1 region as discussed above. The RD1-encoded ESAT-6 protein, which is absent in BCG, is a potent inducer of Th-17 cells which are activated following interactions between ESAT-6 and TLR-2. Improved protective efficacy is seen when BCG is complemented with the ESAT-6 containing RD1 region; and enhanced Th-17 responses appear to be a component of this protection (72) as do antigen non-specific CD8+ and natural killer cells (see above).

B-cell and antibody responses in the context of TB have not been studied to the extent of their T-cell counterparts due to what may turn out to be an under-appreciation of their importance. Recent evidence suggests a role for functionally distinct antibody responses in latent TB infection (73, 74) and antibody and memory B-cell responses in BCG-vaccinated adults have been demonstrated (75, 76) and may correlate with a reduced risk of TB disease in infants (77, 78).

Despite evidence for longevity in the protective efficacy of BCG in some settings, this protection has yet to be attributed to a defined population of memory immune cells. It is difficult in humans to differentiate mycobacteria-specific, long-lived memory cells that are present because of an historic BCG vaccination from responses that are constantly re-stimulated by exposure to environmental mycobacteria. This is easier to study in animal models where exposure to mycobacteria is more easily controlled. In pre-clinical studies of the recombinant BCG vaccine candidate BCG∆ureC:hly in mice, there was an association between vaccine-induced protection and CXCR5+ CCR7+ central memory CD4+ T-cells (79). Also, in mice, the quality of the T-cell response to vaccination was reliant upon the persistence of live BCG. Once drug-sensitive BCG bacteria were removed by drug treatment of mice, there was a reduction in antigen-specific T effector cells, and a reduction in protection (80). In man, BCG is still present in vaccination sites at 1 month after vaccination (50) but data on its longer-term survival are limited. Although there have been case reports of longer-term survival of BCG [i.e., where an HIV-infected individual developed disseminated BCG 30 years after vaccination (81)], disseminated BCG infections have not increased dramatically in countries where HIV infection is more prevalent, suggesting that in most cases BCG does not survive long-term in vaccinees.

### BCG-Induced Trained Innate Immunity

Although interactions between the BCG vaccine and innate immune system receptors such as complement receptor-3 and TLRs 2 and 4 were previously known (82, 83), a more recent discovery is the ability of BCG to bestow a type of immunological memory on innate cells, so-called trained innate immunity (84, 85). Mediated by NOD2 receptor signaling and epigenetic modification of macrophages, the phenomenon is characterized by an enhanced ability of macrophages to produce cytokines such as TNFα and IL-6 in response to unrelated microorganisms and TLR ligands following BCG vaccination. Natural killer cells have also been demonstrated to contribute to the trained innate effect both in humans and in mice (86). Trained innate cells alter their metabolism, switching from oxidative phosphorylation to aerobic glycolysis (87). Trained immunity is reduced if monocytes from controls are stimulated with irradiated BCG bacilli rather than live BCG (88). Trained innate immunity is clearly a prime candidate mechanism to explain the non-specific protective effects of BCG described above. To this end, our group and others have investigated whether BCG vaccination of infants has the same effect on the innate immune response. Jensen et al. in a cohort of West African infants and our group in United Kingdom infants both showed evidence of enhanced innate responses to non-specific stimuli following infant BCG vaccination (89, 90).

## Factors That Affect BCG-Induced Immune Responses

### Vaccination Setting

As noted above BCG vaccination is less effective against pulmonary TB in some settings. This phenomenon has been explored using immunological analysis, which confirmed that >80% of United Kingdom adolescents did not make an IFNγ response to PPD in diluted whole blood cultures before vaccination, whereas over 60% of Malawian adolescents and young adults did; 12 months post-vaccination there was still marked immunological memory present in most of the United Kingdom vaccinees whereas the Malawian vaccinees did not show a vaccine-induced enhancement of their responses (91). Less expected was the finding that Malawian infants, presumed to be immunologically naïve when vaccinated, also made lower IFNγ responses than United Kingdom infants (92) with reduced Th-1 and increased Th-2 cytokine responses (93). In the United Kingdom, BCG-vaccinated infants show increased BCG growth inhibition compared with unvaccinated infants (71); similar studies have not yet been reported for African infants. Again, there are other reports where growth inhibition is not associated with BCG vaccination, or with protection. Although BCG-induced immunity can be long-lived, there was no association between childhood BCG vaccination and mycobacterial growth inhibition in human bronchoaleolar lavage cells from TB contacts as assessed by colony counts (94). In a study of BCG-vaccinated South African infants, tested at 4–6 months of age, there was no significant association between growth inhibition of BCG and subsequent development of disease (77).

A number of other explanations have been suggested for varying immunogenicity and protective efficacy of the BCG vaccine. There are clearly factors related to location or setting. There are seasonal influences that determine how T-cell responses are induced or maintained, as shown for IFNγ responses in Malawi (92). Varying vaccination schedules may affect immunogenicity, as shown in some studies where, when BCG vaccine was given with oral polio vaccine (OPV) in Guinea Bissau, IFNγ, and IL-5 responses to PPD were reduced (95); however, any such immunosuppressive effects resulting from oral live polio vaccination should now reduce as OPV is replaced with inactivated polio vaccine.

### Maternal Factors

In some of these settings, many mothers will be infected with latent TB, and this could lead to either *in utero* sensitization or tolerization to mycobacterial antigens. However, so far LTBI in mothers has only been shown to have a transient effect on responses to mycobacterial antigens in their infants (96, 97). Maternal BCG scar was associated with stronger proinflammatory cytokine responses to innate stimuli in infant cord blood, but not to stronger responses in the mothers themselves (98). Other maternal infections may also modulate BCG-induced immunity. Although infants will not acquire helminth infections until well after BCG vaccination, in LMIC many of their mothers will carry helminth infections. There was no clear association of maternal helminth infection with the infants immune responses to vaccination (99) and treating pregnant women in the second or third trimester with albendazole, praziquantel, or both did not affect infant responses to BCG, tetanus, or measles vaccination (100). Co-infections in the infants themselves could also modulate immunity. Cytomegalovirus infection is acquired early in life by most infants in Africa and has a profound effect on the immune system (101–103). Other factors could include nutrition, genetics, and season of birth (104, 105).

### Delayed BCG Vaccination

Many infants do not receive BCG vaccination immediately after birth, and delays in vaccination might improve immunity, due to maturation of the immune system, which is skewed toward Th-2 T-cell immune responses at birth. However, delaying BCG vaccination has shown less consistent effects on BCG-induced immunogenicity than might have been expected (58, 106–110). Partly this lack of consensus results from variations in the approaches taken by different studies, including the age until which vaccination is delayed, the time points post-vaccination at which blood samples are obtained and analyzed and the range of assays used and immunological functions investigated. Delayed BCG may circumvent the risks associated with at birth vaccination of HIV-exposed infants. Tchakoute et al. reported that immune responses in HIV-exposed, uninfected infants vaccinated at 8 weeks of age were at least as robust as those in infants vaccinated at birth and in some respects (enhanced CD4 T-cell IFNγ responses and increase cytokine functionality in CD4 and CD8 T-cells) delayed vaccination was better (110). Kagina et al. demonstrated better T-cell cytokine responses (including polyfunctional CD4 T-cells) at 1 year in infants where BCG was delayed to 10 weeks of age. The differences detectable at an earlier time point of 10 weeks post-vaccination were more modest which agrees with the findings of Ritz et al. who conducted a similar study (107, 109).

In contrast to the studies mentioned above in which delaying BCG was either not different or in some cases better than vaccination at birth, two further studies reported that immune responses were enhanced in infants who received BCG at birth compared with those in which it was delayed. In the Gambia, Th-1 and Th-17 responses measured 4.5 months after vaccination were reduced when BCG was delayed to 4.5 months (approximately 19 weeks of age) however the differences became less apparent when samples were obtained later, at 9 months of age (106). In contrast Lutwama et al. found that at 9 months of age it was still possible to detect better CD4 and CD8 T-cell cytokine responses in infants vaccinated at birth compared with those vaccinated at 6 weeks of age (108). In addition to the variables mentioned above, the different settings of all these studies should be noted. With the exception of the study by Ritz et al., performed in Australia (109), most of these studies were performed in Africa, although they were located in different settings; some of which (Uganda and the Gambia) were more equatorial than others (South Africa). The same factors that determine the geographical variation in BCG efficacy may also account for differences in the effect of delayed vaccination.

## Toward Correlates of Protection

### T-Cell and B-Cell Immune Responses as Correlates of Protection

Although most of these studies have investigated immunogenicity rather than protection, a large study based in South Africa that recruited 5,726 BCG-vaccinated infants and followed them for 2 years, has enabled correlates of risk of disease to be investigated more directly. In this cohort, the proportion of polyfunctional T-cells in BCG stimulated cultures was not associated with reduced risk of developing TB disease (111). Despite comprehensive analyses using gene expression, correlates of risk could not be identified. The protected and unprotected infants showed marked heterogeneity in gene expression patterns, with distinct subsets in both protected and unprotected groups with higher or lower monocyte:lymphocyte ratios and myeloid or lymphoid cell activation (112) (see below). In the only other BCG-vaccinated infant cohort followed for protection against the development of disease, infants from the MVA85A vaccine trial in which the MVA85A vaccination following BCG had not induced significant efficacy (113) were assessed for biosignatures associated with protection or progression. T-cell activation (CD4 T-cells expressing HLA DR) was associated with increased risk of progression to disease, but BCG-induced IFNγ producing T-cells were associated with reduced risk of TB (77).

Perhaps as a response to the lack of confirmed T-cell correlates of protection induced by BCG vaccination, there is renewed interest in B-cell immunity in TB, which as noted above has been under-studied. Although antibodies have not provided useful diagnostic tests for TB, expression of B-cell-associated genes are modulated during TB treatment (114) and the frequencies of plasmablasts are increased in those with active TB disease compared with healthy community controls, and community controls with evidence of latent TB infection have higher numbers of both plasmablasts and memory B cells (78). Memory B cells specific for PPD were higher in BCG-vaccinated adult donors than unvaccinated subjects, even from individuals vaccinated 13–45 years earlier (75). It is therefore interesting that in infants from the MVA vaccine trial higher concentrations of IgG antibodies to Ag85 at 5–7 months of age were associated with a reduced risk of subsequent TB (77).

### Unbiased Functional Assays of BCG-Induced Protection: Mycobacterial Growth Inhibition

Functional assays that do not rely on prior knowledge of specific immune correlates of protection have been of interest for some time. Such assays can potentially indicate vaccine-induced protection in easily accessible samples (e.g., whole blood or PBMC) and are unbiased in the sense that they do not focus on any given immune component. Early interest in mycobacterial growth inhibition as a surrogate marker of protection involved the *in vitro* expansion of lymphocytes with relevant antigens prior to incubation with BCG-infected monocytes. PBMC previously stimulated with mycobacterial whole cell lysate or live BCG induced infected monocytes to control the growth of BCG better as did previous BCG vaccination of donors themselves (115). In another study using an assay that did not rely on *in vitro* stimulation of PBMC, *ex vivo* whole blood or fresh PBMC from adults who were recently BCG vaccinated (either primary vaccination or re-vaccination) were incubated with live BCG and growth inhibition determined by Bactec MGIT analysis. The study reported that PBMC from adults who had received primary BCG did control mycobacterial growth better *ex vivo* than unvaccinated controls (116). In a study of infants vaccinated in early life (at approximately 7 weeks of age), our group demonstrated better PBMC-mediated growth inhibition of BCG in vaccinated infants compared with unvaccinated controls 3 months after vaccination, although the effect had waned by 12 months (71). Despite these positive data supporting the use of mycobacterial growth inhibition as a surrogate marker of vaccine-induced protection, it should be noted that, as with vaccine-induced T-cell responses, growth inhibition was not associated with reduced risk of TB disease in BCG-vaccinated South African infants (77). The concept of mycobacterial growth inhibition has been taken a step further with human challenge models whereby protection is assessed following the administration of an *in vivo* mycobacterial challenge, usually an intradermal inoculation of live BCG (117, 118). Such an approach has allowed the association of *in vivo* control of live BCG with patterns of gene expression and cellular immune responses (119).

### Variable Profiles of Response to BCG Vaccination: Hampering the Search for Correlates of Protection?

As discussed above, population differences exist in adult and infant immune response to BCG (91–93) however, even within populations, marked heterogeneity in the host response to BCG vaccination has been observed. South African infants who received BCG clustered into two groups of responders that displayed distinct myeloid or lymphoid activation patterns of gene expression (112). Furthermore, Boer and colleagues found that young adults receiving primary BCG vaccination responded in a surprisingly dichotomous manner. Either broadly proimflammatory responses with local reactogenicity and induction of polyfunctional CD4 T-cells or responses characterized by mild local inflammation, poor cytokine, and polyfunctional CD4 T-cell induction and a predominance of regulatory CD8 T-cells were detected (120).

Although there is opportunity in the fact that BCG vaccination demonstrates protective efficacy in infants and in adults in some geographical locations, there are clearly some host-related factors that modify immune responses to BCG and give rise to significant heterogeneity. Until we understand these factors better and take them into account, it will be difficult to identify broadly relevant correlates of protection in BCG-vaccinated cohorts.

## Conclusion

Although there has been much progress over the last 20 years, we are still unable to identify BCG-induced correlates of protection in vaccinated infants. This remains a priority area for further research, as it is possible that the development of effective boosting TB vaccines could be abandoned simply because we have yet to discover the essential aspect of BCG-induced immunity to TB that should be boosted. Similarly, a better understanding of BCG-induced correlates of protection will help novel, live mycobacterial vaccines avoid the pitfalls that have caused BCG to fail in certain settings. To date, the search for BCG-induced correlates has been far too simplistic. However, the advent of more detailed immune and memory cell phenotyping obtained using mass cytometry (121) and the availability of more gene expression datasets in recent times is providing an increasingly nuanced picture. Coupled with sophisticated bioinformatic analyses, these new approaches may soon identify the complex biosignatures associated with BCG-induced protection against TB.

## Author Contributions

HD and SS wrote, edited, and approved the final version of this article.

## Conflict of Interest Statement

The authors declare that the research was conducted in the absence of any commercial or financial relationships that could be construed as a potential conflict of interest.
